# Public awareness, trust, perceived usefulness, and willingness to use ChatGPT for healthcare communication among adults in the Asir Region, Saudi Arabia: a cross-sectional study

**DOI:** 10.3389/fpubh.2026.1862006

**Published:** 2026-08-21

**Authors:** Geetha Kandasamy, Khalid Orayj, Rayah Asiri, Asma M. Alshahrani, Ghaleb Alharbi, Sarah A. Abutaily, Atheer Yahya Al Suhaym, Atheer Ali Alyahya

**Affiliations:** 1 Department of Clinical Pharmacy, College of Pharmacy, King Khalid University, Abha, Saudi Arabia; 2 Department of Clinical Pharmacy, College of Pharmacy, Shaqra University, Dawadimi, Saudi Arabia; 3 Department of Pharmacy Service, Prince Sultan Military Medical City, Riyadh, Saudi Arabia; 4 Department of Emergency, Eradah Hospital for Mental Health in Jazan, Jazan, Saudi Arabia; 5 Department of Neurology, Prince Sultan Military Medical City (PSMMC), Riyadh, Saudi Arabia

**Keywords:** artificial intelligence, ChatGPT, healthcare communication, perceived usefulness, public awareness, Saudi Arabia, trust

## Abstract

**Background:**

Artificial intelligence (AI) is increasingly used in healthcare, with tools such as ChatGPT, which enhances access to health information. Understanding public awareness, trust, and perceived usefulness, along with demographic influences, is essential for responsible AI adoption. This study aimed to evaluate awareness, trust, and perceived usefulness of ChatGPT in healthcare communication among adults in the Asir Region, Saudi Arabia.

**Methods:**

A cross-sectional study was conducted among adults (≥18 years) in the Asir Region from November 2025–January 2026. A validated Arabic questionnaire assessed sociodemographics, prior ChatGPT use, awareness, trust, perceived usefulness, risks, and willingness. Data were analyzed using descriptive statistics and multiple linear regression to identify predictors of willingness (*p* < 0.05).

**Results:**

A total of 429 adults participated in the study. Most participants reported prior use of ChatGPT (70.40%). Awareness of ChatGPT and AI in healthcare was moderate, with higher scores among younger and more educated participants (*p* < 0.05). Participants generally reported favorable perceptions of the usefulness of ChatGPT, particularly for patient education and access to information, although trust remained moderate. Perceived risks included misinformation, privacy concerns, and overreliance. Overall, 61.77% of participants were willing to use ChatGPT, with significant variation by age (*p* = 0.030). Regression analysis showed that higher awareness (*β* = 0.41, *p* < 0.001) and trust (*β* = 0.36, *p* < 0.001) were positively associated with willingness, while risk perception was negatively associated (*β* = −0.12, *p* = 0.019). Gender and education were not statistically significant predictors.

**Conclusion:**

This study highlights generally high awareness and positive perceptions of ChatGPT among adults in the Asir Region of Saudi Arabia, with moderate levels of trust and perceived usefulness and concerns related to misinformation, privacy, and safety. Higher awareness and greater trust and perceived usefulness were associated with increased willingness to use ChatGPT, while perceived risks were associated with lower willingness to use ChatGPT. Variations across age and educational levels indicate differences in familiarity and confidence in AI tools. Overall, the findings reflect cautious but favorable openness toward AI in healthcare and emphasize the need for improved AI literacy, trust-building strategies, and regulatory oversight to support safe and informed engagement with generative AI technologies. The use of convenience sampling may limit the generalizability of the findings.

## 1 Introduction

Artificial intelligence (AI) driven chatbots, often referred to as conversational agents, are computer programs designed to emulate human interaction through text, speech, or visual communication channels (1, 2). With the rapid advancement of digital technology and widespread access to the internet, smartphones, and computers, AI chatbots have become increasingly relevant tools for providing convenient, autonomous, and engaging health information and services. Among these, ChatGPT, a generative language model developed by OpenAI, has gained global attention for its ability to simulate human-like dialogue, interpret complex questions, and deliver contextually appropriate responses. These features highlight its potential role in supporting health communication and patient education across diverse populations (3).

AI chatbots address many of these limitations by offering on-demand accessibility, personalized interaction, and sustained engagement over time. Their conversational adaptability allows individuals, particularly those who may experience stigma or discomfort when seeking medical care, to communicate privately and safely from any location at any time (4, 5). During large-scale public health emergencies, conventional communication channels can become overloaded, hindering the timely delivery of accurate information. In such situations, digital health solutions have emerged as vital tools for bridging communication gaps by providing scalable, real-time, and dependable information pathways (6). Within this context, AI-based chatbots have demonstrated a strong capacity to manage large volumes of public inquiries efficiently and deliver critical health information continuously throughout the day (7).

Despite early skepticism regarding reliability, bias, and ethical concerns (8, 9), advancements in AI architecture and natural language processing have substantially improved the accuracy, contextual understanding, and perceived dependability of chatbot systems. Recent evidence indicates that AI chatbots can effectively handle substantial volumes of real-time user interactions and provide consistent, evidence-based responses (10). Their integration into healthcare environments has been associated with measurable benefits, including reduced workload for medical personnel and improved patient outcomes through faster, more reliable information exchange (11). Moreover, the introduction of advanced language models such as GPT-4 has markedly enhanced the ability of chatbots to understand complex queries, respond empathetically, and adapt to diverse communication contexts, thereby strengthening user confidence and trust (12).

These technological developments underscore the growing usefulness of ChatGPT as a tool for healthcare delivery and patient engagement. However, public acceptance remains variable. While many users perceive ChatGPT as useful for accessing health information, concerns persist regarding accuracy, bias, privacy, and overreliance on AI-generated advice (13). A recent Saudi study involving 581 participants from medical, information technology, and general-public backgrounds reported generally positive attitudes toward AI in healthcare, although ethical concerns, liability, and patient safety remained prominent. Participants emphasized the importance of physician oversight when AI is used in healthcare settings (14). These concerns are important because trust is a known predictor of intention to use AI health technologies, including ChatGPT (15).

Evidence from Saudi Arabia shows generally positive perceptions of ChatGPT and AI-based healthcare tools, although concerns related to trust, privacy, and reliability persist (16, 17). Saudi Arabia has also made substantial progress in digital health transformation under Vision 2030, including expansion of telemedicine, electronic health records, and digital health platforms to improve accessibility and efficiency (18–20). Despite this progress, privacy and data security continue to influence public trust and acceptance of healthcare technologies in Saudi Arabia (21). Similarly, across the GCC region, including the United Arab Emirates, Oman, and Bahrain, national digital health systems have been widely implemented, although challenges remain in interoperability, governance, and standardization (22–27).

The United Arab Emirates is a regional leader in AI adoption in healthcare, integrating AI into diagnostics, decision support, and service optimization as part of national digital transformation strategies (28). However, across the GCC, structural and operational limitations may still affect the adoption of emerging generative AI tools such as ChatGPT (26, 27, 29–31). Although awareness of AI-based health technologies is increasing, concerns regarding privacy, reliability, and misinformation continue to influence acceptance. Digital health literacy plays a key role in shaping individuals’ ability to evaluate and use digital health information (32, 33), while trust in healthcare systems strongly predicts adoption of AI-driven tools (34). However, research in Arab populations remains limited, particularly regarding generative AI, where cultural and contextual factors may further influence acceptance.

Despite rapid digital transformation in Saudi Arabia and the GCC, evidence on public perceptions of generative AI tools such as ChatGPT remains limited, particularly among the general population. The Asir Region, with its mix of urban and rural communities, provides an important setting to examine public perceptions of ChatGPT, including trust, usefulness, and willingness to use it in healthcare communication.

Therefore, this study aims to evaluate public awareness, trust, perceived usefulness, perceived risk, and willingness to use ChatGPT for healthcare communication among adults in the Asir Region, Saudi Arabia. This study is grounded in an extended Technology Acceptance Model (TAM) integrated with the Unified Theory of Acceptance and Use of Technology (UTAUT) and trust-based AI adoption frameworks (35–43). TAM explains technology acceptance through perceived usefulness and perceived ease of use (35–37), while UTAUT extends this with performance expectancy, effort expectancy, social influence, and facilitating conditions (37–39). In healthcare contexts, these models are often extended by incorporating trust and perceived risk, as AI adoption depends not only on perceived benefits but also on confidence in system reliability and safety (40–43).

## 2 Methods

### 2.1 Study design

This research employed a cross-sectional, descriptive study design to assess public awareness, trust, and perceived usefulness of ChatGPT in healthcare among adults residing in the Asir Region, Saudi Arabia.

### 2.2 Study setting and duration

The study was conducted across various cities and communities in the Asir Region, located in the southern part of Saudi Arabia. Data collection was carried out between November 2025–January 2026.

### 2.3 Study population and sample size determination

The target population included Saudi and non-Saudi adults residing in the Asir Region. Participants were recruited using a non-probability convenience sampling technique through online dissemination on social media platforms (e.g., WhatsApp, Twitter, Telegram) to facilitate broad community participation and rapid data collection. The minimum required sample size was calculated using Raosoft’s online sample size calculator, assuming a 95% confidence level, a 5% margin of error, and an estimated response distribution of 50%. Based on the estimated total population of the Asir Region (approximately 2.0–2.05 million), as reported by the General Authority for Statistics (GASTAT), the required sample size was 384 participants. A total of 443 survey entries were recorded. Of these, 8 participants did not provide electronic informed consent and did not proceed to the questionnaire. An additional 6 responses were excluded due to incomplete data. Therefore, the final analytic sample consisted of 429 participants.

For contextual interpretation, the estimated adult population (≥18 years) of the Asir Region is approximately 1.2–1.35 million based on national age-structure distributions. The study sample (*n* = 429) therefore represents a small proportion of the overall adult population. The use of online convenience sampling may introduce selection bias and limit generalizability; however, it is widely used for rapid community-based and digital health perception studies.

### 2.4 Data collection tool

The instrument was developed based on a review of previously conducted studies on artificial intelligence and chatbot applications in healthcare (44–47) and comprised six main sections. Section A included the informed consent statement, which outlined the study objectives, voluntary participation, and assurances of anonymity and confidentiality. Section B gathered sociodemographic information such as age, gender, marital status, education, occupation, monthly income, nationality, and place of residence, as well as prior exposure to ChatGPT or other AI-based tools. Section C assessed participants’ awareness and knowledge of ChatGPT and artificial intelligence applications in healthcare through four items measured on a five-point Likert scale ranging from strongly disagree (1) to strongly agree (5). Section D evaluated trust and perceived usefulness of ChatGPT in healthcare communication and patient education using six items on the same Likert scale. Section E explored perceived risks and ethical concerns through five items addressing issues such as misinformation, data privacy, and potential reductions in human interaction between patients and healthcare providers. Finally, Section F examined participants’ willingness and future intention to use ChatGPT for health-related purposes using five-point Likert responses.

### 2.5 Scoring of composite variables

All items in Sections C–F were rated on a 5-point Likert scale ranging from 1 (strongly disagree) to 5 (strongly agree). Composite scores were calculated by averaging item responses within each domain to facilitate comparison across constructs. The awareness score was derived from four items and ranged from 1 to 5, with higher scores indicating greater awareness and knowledge of ChatGPT and AI in healthcare. The trust and perceived usefulness score was derived from six items and ranged from 1 to 5, with higher scores indicating greater trust in ChatGPT and perceived usefulness in healthcare settings. The perceived risk score was derived from five items and ranged from 1 to 5, with higher scores indicating greater concern regarding ethical, privacy, and safety issues associated with ChatGPT use. The willingness score was derived from four items and ranged from 1 to 5, with higher scores indicating greater willingness to use, recommend, and support the integration of ChatGPT into healthcare. All composite scores were treated as continuous variables in the statistical analyses.

### 2.6 Questionnaire validation and pilot testing

The questionnaire was initially developed in English and subjected to expert validation to ensure content accuracy, relevance, and clarity. It was then translated into Arabic and subsequently back-translated into English to verify semantic equivalence and ensure that the meaning of each item remained consistent across both language versions. Content validity was assessed through expert panel review for relevance, clarity, and cultural appropriateness. The experts independently evaluated the items and provided qualitative feedback to refine wording and improve clarity. Formal quantitative content validity indices (CVI) were not calculated; however, expert consensus was used to refine and finalize the instrument.

The awareness, trust/perceived usefulness, perceived risk, and willingness domains were developed based on previously published studies. These were adapted for the current context rather than being used as pre-validated standardized instruments. Content validity was established through expert review, and internal consistency reliability was assessed using Cronbach’s alpha during pilot testing. An expert panel of five specialists reviewed the instrument for conceptual alignment, linguistic clarity, and cultural appropriateness. The panel included two professors of clinical pharmacy, one expert in medical informatics, one public health researcher, and one biostatistician. Based on their feedback, minor modifications were made to refine the wording and improve item comprehensibility.

Following validation, a pilot test was conducted among 30 participants to evaluate the questionnaire’s reliability and comprehensibility. Internal consistency reliability was assessed using Cronbach’s alpha for the Likert-scale sections. The awareness subscale (4 items) demonstrated acceptable reliability with an alpha coefficient of 0.74, while the trust and perceived risk subscales showed good internal consistency with alpha values of 0.82 and 0.84, respectively. These findings confirm that the instrument possessed satisfactory content validity, linguistic accuracy, and internal reliability for assessing public perceptions of ChatGPT in healthcare.

### 2.7 Participant recruitment, eligibility, consent, and data confidentiality

Participants were recruited through an open online survey link distributed via social media platforms, including WhatsApp, Twitter (X), and Telegram, targeting community groups within the Asir Region, Saudi Arabia. Participation was voluntary, and no incentives were provided.

Participants were eligible if they were adults aged ≥18 years, residing in the Asir Region, and able to read Arabic. Responses that were incomplete or duplicated were excluded from the final analysis. Before accessing the questionnaire, participants were presented with an information page describing the study objectives, voluntary nature of participation, and confidentiality assurances. Electronic informed consent was obtained by requiring participants to actively agree before proceeding to the survey. Only participants who provided consent were allowed to continue. No personally identifiable information (including names, phone numbers, email addresses, IP addresses, or device identifiers) was collected or stored. All responses were recorded anonymously and analyzed in aggregate form. Survey data were downloaded from Google Forms and stored in password-protected, encrypted files on a secure institutional device. Access to the dataset was restricted exclusively to the principal investigator and authorized research team members.

### 2.8 Data analysis

Data were analyzed using IBM SPSS Statistics version 28.0. Descriptive statistics, including frequencies, percentages, means, and standard deviations, were used to summarize sociodemographic characteristics and study variables. Normality of continuous variables was assessed using the Shapiro–Wilk test. Missing data were minimal and handled using complete-case analysis (listwise deletion), and only fully completed responses (n = 429) were included in the final analysis. No imputation methods were applied. Multicollinearity among independent variables was assessed using variance inflation factor (VIF) and tolerance values. Bivariate associations between sociodemographic variables and study outcomes (awareness, trust, and willingness) were examined using independent sample t-tests for binary variables and one-way analysis of variance (ANOVA) for variables with more than two groups. Effect sizes were reported using eta squared (η^2^) for ANOVA and Cohen’s d for t-tests. Multiple linear regression analysis was performed to identify predictors of willingness to use ChatGPT in healthcare. Awareness, trust, perceived risk, and selected sociodemographic variables (age, gender, and education) were entered simultaneously into the model. Regression coefficients (*β*), standard errors, 95% confidence intervals, and *p*-values were reported. Model fit was evaluated using R^2^ and adjusted R^2^, and model assumptions were assessed using the Durbin–Watson statistic and VIF. A p-value of <0.05 was considered statistically significant.

### 2.9 Ethical considerations

Ethical approval for this study was obtained from the Institutional Research Ethics Committee of King Khalid University (HAPO-06-B-001; KKU-134-2025-31). The study was conducted in accordance with the Declaration of Helsinki. Electronic informed consent was obtained from all participants prior to participation, and participation was voluntary with the right to withdraw at any time without any consequences. Participant anonymity and confidentiality were strictly maintained. Data were securely stored in encrypted, password-protected files on a secure institutional device with restricted access to the research team. Duplicate responses were minimized using built-in Google Forms response controls and manual screening during data cleaning to ensure data integrity. No financial incentives were provided to participants.

## 3 Results

A total of 429 adults participated in the study. The sociodemographic characteristics of the participants are presented in Table 1. Regarding age distribution, 140 participants (32.63%) were aged 18–29 years, 87 (20.28%) were 30–39 years, 87 (20.28%) were 40–49 years, and 115 (26.81%) were aged ≥50 years. In terms of gender, 291 participants (67.83%) were male and 138 (32.17%) were female. For marital status, 249 participants (58.04%) were married, while 180 (41.96%) were single, divorced, or widowed. With respect to educational level, 174 participants (40.56%) were graduates, 137 (31.93%) held a diploma, 95 (22.14%) had secondary school education, and 23 (5.36%) had high school education. Regarding occupation, 164 participants (38.23%) were employed, 117 (27.27%) were students, 80 (18.65%) were unemployed or homemakers, and 68 (15.85%) were retired. Monthly income distribution showed that 71 participants (16.55%) earned <5,000 SAR, 45 (10.49%) earned 5,000–10,000 SAR, 99 (23.08%) earned 10,001–15,000 SAR, 114 (26.57%) earned >15,000 SAR, and 100 (23.31%) reported not applicable income. Regarding nationality, 372 participants (86.71%) were Saudi and 57 (13.29%) were non-Saudi. Place of residence was urban for 306 participants (71.33%) and rural for 123 (28.67%). Finally, 302 participants (70.40%) reported having used ChatGPT, while 127 (29.60%) reported not using ChatGPT.

**Table 1:** Sociodemographic characteristics of participants.

Variable	Category	*N* (*n* = 429)	Percentage (%)
Age (years)	18–29	140	32.63
	30–39	87	20.28
	40–49	87	20.28
	≥50	115	26.81
Gender	Male	291	67.83
	Female	138	32.17
Marital status	Married	249	58.04
	Single/divorced/widowed	180	41.96
Educational level	Graduates	174	40.56
	Diploma	137	31.93
	Secondary school	95	22.14
	High school	23	5.36
Occupation	Employed	164	38.23
	Student	117	27.27
	Unemployed/homemaker	80	18.65
	Retired	68	15.85
Monthly income (SAR)	<5,000	71	16.55
	5,000–10,000	45	10.49
	10,001–15,000	99	23.08
	>15,000	114	26.57
	Not applicable	100	23.31
Nationality	Saudi	372	86.71
	Non-Saudi	57	13.29
Place of residence	Urban	306	71.33
	Rural	123	28.67
Used ChatGPT	Yes	302	70.40
	No	127	29.60

Table 2 summarizes participants’ awareness, knowledge, trust, and perceived usefulness of ChatGPT and artificial intelligence in healthcare. For the statement “I understand what ChatGPT or similar AI chatbots are,” 17 participants (3.96%) strongly disagreed, 40 (9.32%) disagreed, 70 (16.32%) were neutral, 183 (42.66%) agreed, and 119 (27.74%) strongly agreed, with a mean score of 3.81 ± 1.07. For the statement “AI technologies are already used in Saudi healthcare,” 35 participants (8.16%) strongly disagreed, 67 (15.62%) disagreed, 124 (28.90%) were neutral, 139 (32.40%) agreed, and 64 (14.92%) strongly agreed, with a mean score of 3.30 ± 1.15. For the statement “I have received education/training about AI,” 99 participants (23.08%) strongly disagreed, 137 (31.93%) disagreed, 93 (21.68%) were neutral, 72 (16.78%) agreed, and 28 (6.53%) strongly agreed, with a mean score of 2.52 ± 1.20. For the statement “I can differentiate ChatGPT from other chatbots,” 46 participants (10.72%) strongly disagreed, 80 (18.65%) disagreed, 125 (29.14%) were neutral, 128 (29.84%) agreed, and 50 (11.66%) strongly agreed, with a mean score of 3.13 ± 1.17. Perceived usefulness was generally positive, particularly for supportive and educational roles. The highest agreement was for ChatGPT supports patient education (strongly agree/agree = 41.26% + 26.81% = 68.07%, mean = 3.80 ± 1.02) and ChatGPT improves access to information (41.03% + 27.04% = 68.07%, mean = 3.79 ± 1.04). A similarly high level of agreement was observed for ChatGPT assists healthcare professionals (40.09% agree, 21.68% strongly agree; mean = 3.64 ± 1.08) and helps patients understand conditions (38.69% agree, 21.68% strongly agree; mean = 3.62 ± 1.07). Perceptions related to accuracy and safety were comparatively moderate. For ChatGPT provides accurate health information, 40.32% agreed or strongly agreed, while 26.34% disagreed or strongly disagreed (mean = 3.17 ± 1.12). Trust in safety showed the lowest positive response among items, with 35.67% agreeing or strongly agreeing that ChatGPT provides safe advice, while 30.77% disagreed or strongly disagreed (mean = 3.06 ± 1.14).

**Table 2:** Participants’ awareness, knowledge, trust, and perceived usefulness of ChatGPT and artificial intelligence in healthcare (*n* = 429).

Statement	Strongly disagree *n* (%)	Disagree *n* (%)	Neutral *n* (%)	Agree *n* (%)	Strongly Agree *n* (%)	Mean ± SD
I understand what ChatGPT or similar AI chatbots are	17 (3.96)	40 (9.32)	70 (16.32)	183 (42.66)	119 (27.74)	3.81 ± 1.07
AI technologies are already used in Saudi healthcare	35 (8.16)	67 (15.62)	124 (28.90)	139 (32.40)	64 (14.92)	3.30 ± 1.15
I have received education/training about AI	99 (23.08)	137 (31.93)	93 (21.68)	72 (16.78)	28 (6.53)	2.52 ± 1.20
I can differentiate ChatGPT from other chatbots	46 (10.72)	80 (18.65)	125 (29.14)	128 (29.84)	50 (11.66)	3.13 ± 1.17

Trust and perceived usefulness of ChatGPT in healthcare (*n* = 429)	Strongly disagree *n* (%)	Disagree *n* (%)	Neutral *n* (%)	Agree *n* (%)	Strongly Agree *n* (%)	Mean ± SD
ChatGPT provides accurate health information	38 (8.86)	75 (17.48)	143 (33.33)	124 (28.90)	49 (11.42)	3.17 ± 1.12
I trust ChatGPT to provide safe advice	43 (10.02)	89 (20.75)	144 (33.57)	105 (24.48)	48 (11.19)	3.06 ± 1.14
ChatGPT assists healthcare professionals	20 (4.66)	45 (10.49)	99 (23.08)	172 (40.09)	93 (21.68)	3.64 ± 1.08
ChatGPT helps patients understand conditions	17 (3.96)	50 (11.66)	103 (24.01)	166 (38.69)	93 (21.68)	3.62 ± 1.07
ChatGPT improves access to information	15 (3.50)	38 (8.86)	84 (19.58)	176 (41.03)	116 (27.04)	3.79 ± 1.04
ChatGPT supports patient education	14 (3.26)	35 (8.16)	88 (20.51)	177 (41.26)	115 (26.81)	3.80 ± 1.02

Participants’ Perceived Risks, Ethical Concerns, and Willingness for Future Use of ChatGPT in Healthcare Table 3. A high level of agreement was observed for the need for regulations, where 177 (41.26%) agreed and 168 (39.16%) strongly agreed, with a mean score of 4.10 ± 0.95. Concerns regarding overreliance weakening judgment were also prominent, with 166 (38.69%) agreeing and 116 (27.04%) strongly agreeing (mean = 3.76 ± 1.07). For the statement that ChatGPT may provide misleading information, 161 (37.53%) agreed and 118 (27.51%) strongly agreed, with a mean of 3.72 ± 1.12. Similarly, 159 (37.06%) agreed and 114 (26.57%) strongly agreed that ChatGPT may compromise data privacy, with a mean of 3.72 ± 1.08. Perceptions that ChatGPT may reduce human interaction showed relatively lower agreement, with 138 (32.17%) agreeing and 96 (22.38%) strongly agreeing, and a mean score of 3.51 ± 1.16. Overall, responses indicate consistent recognition of ethical and practical concerns, particularly regarding regulation, overreliance, and information reliability. For the statement “Willing to use ChatGPT,” 30 participants (6.99%) strongly disagreed, 50 (11.66%) disagreed, 84 (19.58%) were neutral, 151 (35.20%) agreed, and 114 (26.57%) strongly agreed, with a mean score of 3.63 ± 1.11. For “Would recommend ChatGPT,” 27 participants (6.29%) strongly disagreed, 47 (10.96%) disagreed, 89 (20.75%) were neutral, 155 (36.13%) agreed, and 111 (25.87%) strongly agreed, with a mean score of 3.64 ± 1.09. For “Should be integrated into healthcare,” 34 participants (7.93%) strongly disagreed, 59 (13.75%) disagreed, 107 (24.94%) were neutral, 143 (33.33%) agreed, and 86 (20.05%) strongly agreed, with a mean score of 3.44 ± 1.12. For “Likely to use again,” 25 participants (5.83%) strongly disagreed, 46 (10.72%) disagreed, 86 (20.05%) were neutral, 157 (36.60%) agreed, and 115 (26.81%) strongly agreed, with a mean score of 3.68 ± 1.15.

**Table 3:** Participants’ perceived risks, ethical concerns, and willingness for future use of ChatGPT in healthcare (*n* = 429).

Statement	Strongly disagree *n* (%)	Disagree *n* (%)	Neutral *n* (%)	Agree *n* (%)	Strongly agree *n* (%)	Mean ± SD
May provide misleading information	21 (4.90)	46 (10.72)	83 (19.35)	161 (37.53)	118 (27.51)	3.72 ± 1.12
May compromise data privacy	18 (4.20)	43 (10.02)	95 (22.14)	159 (37.06)	114 (26.57)	3.72 ± 1.08
May reduce human interaction	26 (6.06)	61 (14.22)	108 (25.17)	138 (32.17)	96 (22.38)	3.51 ± 1.16
Overreliance weakens judgment	16 (3.73)	40 (9.32)	91 (21.21)	166 (38.69)	116 (27.04)	3.76 ± 1.07
Regulations are needed	9 (2.10)	20 (4.66)	55 (12.82)	177 (41.26)	168 (39.16)	4.10 ± 0.95

willingness and future use of ChatGPT in healthcare (*n* = 429)	Strongly Disagree *n* (%)	Disagree *n* (%)	Neutral *n* (%)	Agree *n* (%)	Strongly agree *n* (%)	Mean ± SD
Willing to use ChatGPT	30 (6.99)	50 (11.66)	84 (19.58)	151 (35.20)	114 (26.57)	3.63 ± 1.11
Would recommend ChatGPT	27 (6.29)	47 (10.96)	89 (20.75)	155 (36.13)	111 (25.87)	3.64 ± 1.09
Should be integrated into healthcare	34 (7.93)	59 (13.75)	107 (24.94)	143 (33.33)	86 (20.05)	3.44 ± 1.12
Likely to use again	25 (5.83)	46 (10.72)	86 (20.05)	157 (36.60)	115 (26.81)	3.68 ± 1.15

Table 4 presents the results of multiple linear regression analysis predicting willingness to use ChatGPT in healthcare (n = 429). Awareness score was positively associated with willingness to use ChatGPT (*B* = 0.384, SE = 0.052, *β* = 0.41, 95% CI: 0.282–0.486, *p* < 0.001, VIF = 1.52). Trust score also showed a positive association (*B* = 0.327, SE = 0.049, *β* = 0.36, 95% CI: 0.231–0.423, p < 0.001, VIF = 1.64). Risk perception was negatively associated with willingness to use ChatGPT (*B* = −0.108, SE = 0.046, *β* = −0.12, 95% CI: −0.198 to −0.018, *p* = 0.019, VIF = 1.21). Age showed a positive association (*B* = 0.118, SE = 0.044, *β* = 0.14, 95% CI: 0.032–0.204, *p* = 0.008, VIF = 1.37). Gender (*B* = 0.041, SE = 0.055, *β* = 0.03, 95% CI: −0.067–0.149, *p* = 0.457, VIF = 1.12) and education (*B* = 0.086, SE = 0.048, *β* = 0.09, 95% CI: −0.008–0.180, *p* = 0.072, VIF = 1.29) were not significantly associated with willingness to use ChatGPT in the multivariable model. The overall model explained 18.2% of the variance in willingness to use ChatGPT (R^2^ = 0.182; adjusted R^2^ = 0.167), with a statistically significant model fit (*F* = 12.87, df = 6, 422, *p* < 0.001). The Durbin–Watson statistic was 1.94, and the maximum VIF value was 1.64, indicating no evidence of multicollinearity.

**Table 4:** Multiple linear regression analysis identifying predictors of willingness to use ChatGPT in healthcare and model diagnostics (*n* = 429).

Predictor	*B*	SE	Standardized *β*	95% CI	*p*-value	VIF
Awareness score	0.384	0.052	0.41	0.282–0.486	<0.001	1.52
Trust score	0.327	0.049	0.36	0.231–0.423	<0.001	1.64
Risk perception	−0.108	0.046	−0.12	−0.198–-0.018	0.019	1.21
Age	0.118	0.044	0.14	0.032–0.204	0.008	1.37
Gender	0.041	0.055	0.03	−0.067–0.149	0.457	1.12
Education	0.086	0.048	0.09	−0.008–0.180	0.072	1.29
Model diagnostics						
R^2^	0.182					
Adjusted R^2^	0.167					
F-statistic (df)	12.87 (6,422)				<0.001	
Durbin-Watson	1.94					
Maximum VIF	1.64					

B, unstandardized regression coefficient; SE, standard error; β, standardized regression coefficient; CI, confidence interval; VIF, variance inflation factor; df, degrees of freedom.

Table 5 shows that awareness, trust and perceived usefulness, and willingness scores varied significantly across age and education groups. Participants aged 18–29 years reported the highest mean scores for awareness (3.74 ± 0.68), trust and perceived usefulness (3.49 ± 0.72), and willingness (3.77 ± 0.81), while those aged ≥50 years reported lower scores for awareness (3.12 ± 0.82), trust (3.01 ± 0.81), and willingness (3.34 ± 0.88), with statistically significant differences observed for awareness (*p* = 0.001), trust (*p* < 0.001), and willingness (*p* = 0.030). No statistically significant differences were observed between males and females for awareness (*p* = 0.412), trust and perceived usefulness (*p* = 0.365), or willingness (*p* = 0.488), with comparable mean scores across both groups. Regarding education, graduate/postgraduate participants had higher mean scores for awareness (3.62 ± 0.71) and trust and perceived usefulness (3.43 ± 0.74) compared to diploma and high school/secondary groups, with statistically significant differences for awareness (*p* = 0.030) and trust (p = 0.030), while differences in willingness were not statistically significant (*p* = 0.092).

**Table 5:** Bivariate associations between sociodemographic variables and study outcomes.

Variable	Awareness Mean ± SD	*p*-value	Trust Mean ± SD	*p*-value	Willingness Mean ± SD	*p*-value
18–29 years	3.74 ± 0.68		3.49 ± 0.72		3.77 ± 0.81	
30–39 years	3.45 ± 0.73		3.32 ± 0.75		3.58 ± 0.83	
40–49 years	3.28 ± 0.77		3.19 ± 0.78		3.49 ± 0.86	
≥50 years	3.12 ± 0.82	0.001	3.01 ± 0.81	<0.001	3.34 ± 0.88	0.030
Male	3.39 ± 0.78		3.24 ± 0.79		3.57 ± 0.84	
Female	3.34 ± 0.80	0.412	3.19 ± 0.82	0.365	3.52 ± 0.86	0.488
High school/secondary	3.16 ± 0.83		3.08 ± 0.82		3.43 ± 0.87	
Diploma	3.38 ± 0.76		3.21 ± 0.79		3.57 ± 0.84	
Graduate/postgraduate	3.62 ± 0.71	0.030	3.43 ± 0.74	0.030	3.69 ± 0.82	0.092

Effect size analyses and additional regression assumption testing results are presented in the Supplementary file. Age showed small-to-moderate effects on awareness (η^2^ = 0.061), trust and perceived usefulness (η^2^ = 0.074), and willingness to use ChatGPT (η^2^ = 0.034). Education demonstrated small effects on awareness (η^2^ = 0.041) and trust/perceived usefulness (η^2^ = 0.038), with a negligible effect on willingness (η^2^ = 0.018). Gender and place of residence showed negligible effects across all outcomes. Prior ChatGPT use demonstrated the largest associations, with moderate-to-large effects on awareness (Cohen’s d = 0.67), trust and perceived usefulness (Cohen’s d = 0.59), and willingness to use ChatGPT (Cohen’s d = 0.73). Regression assumption testing indicated that the model assumptions were adequately met, with normally distributed residuals (Shapiro–Wilk *p* = 0.087), independent errors (Durbin–Watson = 1.94), and no evidence of problematic multicollinearity (VIF range: 1.12–1.64).

## 4 Discussion

This study examined public awareness, trust, perceived usefulness, and willingness to use ChatGPT for healthcare communication among adults in the Asir region of Saudi Arabia, highlighting the influence of demographic and perceptual factors on AI acceptance. Overall, awareness of ChatGPT was generally high, although formal education or training in AI applications remained limited (48). This finding is consistent with previous studies indicating that while exposure to AI technologies is increasing, detailed understanding of their healthcare applications remains relatively low (48, 49). Younger and more educated participants demonstrated higher awareness levels, likely reflecting differences in digital literacy, access to technology, and exposure to emerging digital tools (50, 51).

Higher educational attainment was also associated with greater awareness and trust, which may reflect a better understanding of AI capabilities and limitations. These findings are consistent with evidence suggesting that reliability, transparency, and data security are important determinants of trust in AI-driven healthcare systems (52, 53). Gender was not significantly associated with awareness, trust, perceived usefulness, or willingness, suggesting that familiarity and understanding of AI technologies may play a more important role than demographic characteristics in shaping attitudes toward AI-based healthcare tools (54). Although age was positively associated with willingness to use ChatGPT, its effect was modest, indicating that perceptual factors may be more influential than demographic characteristics in determining acceptance (55).

Participants generally perceived ChatGPT as useful for improving access to health information and supporting patient education, although trust in the reliability and accuracy of AI-generated information remained more cautious. This pattern suggests that users may recognize the practical benefits of ChatGPT while remaining uncertain about its suitability for healthcare decision-making. Similar findings have been reported among healthcare professionals in Saudi Arabia, where ChatGPT was viewed as valuable for literature appraisal, patient support, and research assistance but less suitable for independent clinical decision-making (56). Studies from Taiwan have likewise shown that perceived usefulness is strongly associated with AI adoption, while trust plays a central role in shaping attitudes toward AI tools (57). The observed associations between trust, perceived usefulness, age, and education further suggest that individual characteristics influence perceptions of AI utility and safety (57, 58).

Participants also expressed concerns regarding misinformation, privacy breaches, reduced human interaction, and overreliance on AI systems that could affect clinical judgment (59–61). The consistency of these concerns across demographic groups suggests broad awareness of the potential risks associated with AI use in healthcare. Consistent with previous research, participants emphasized the need for regulatory frameworks and safeguards to support the safe and responsible deployment of AI technologies (59, 60). A recent multinational study similarly reported generally positive attitudes toward healthcare AI chatbots while highlighting the importance of trust, transparency, and governance in supporting their adoption (62).

Regression analysis identified awareness and trust/perceived usefulness as the strongest predictors of willingness to use ChatGPT, whereas risk perception showed a negative association (58, 63). Awareness emerged as the strongest predictor of willingness, likely because familiarity with ChatGPT reduces uncertainty and improves confidence in its use. Increased exposure to AI tools may also enhance perceived behavioral control and reduce concerns about misuse, thereby facilitating greater acceptance (64). Trust was the second strongest predictor of willingness, likely reflecting perceived safety and reliability and compensating for limited clinical understanding of AI systems (65). In contrast, the negative association between risk perception and willingness may reflect concerns about misinformation, inaccurate outputs, and the potential for clinical errors when AI-generated information is applied in healthcare settings. This finding is consistent with broader skepticism toward AI in healthcare, where perceived risks can act as barriers to adoption despite recognized benefits (66–69). Together, these findings suggest that familiarity and confidence in AI technologies are more influential determinants of willingness than demographic characteristics alone (52, 58).

The regression model explained a modest proportion of variance in willingness to use ChatGPT (R^2^ = 0.182; adjusted R^2^ = 0.167), indicating that although awareness, trust, and risk perception are significant factors, a substantial proportion of variability remains unexplained. This suggests that willingness to use AI-based healthcare tools is likely influenced by additional psychological, social, and contextual factors, including prior experience with digital health technologies, perceived behavioral control, technology readiness, and perceived usefulness in specific healthcare scenarios. Furthermore, the cross-sectional design and reliance on self-reported measures may limit the ability to capture the complexity of actual behavior and causal relationships.

Therefore, the findings should be interpreted as identifying important factors associated with willingness rather than providing a comprehensive predictive model. The results underscore the importance of targeted public engagement, AI literacy initiatives, and transparent communication regarding the capabilities and limitations of AI systems. Efforts to strengthen reliability, explainability, privacy protection, and regulatory oversight may further enhance public trust and support the responsible integration of AI tools into healthcare communication and patient education (59, 61).

### 4.1 Practical and policy implications

**Figure 1:**
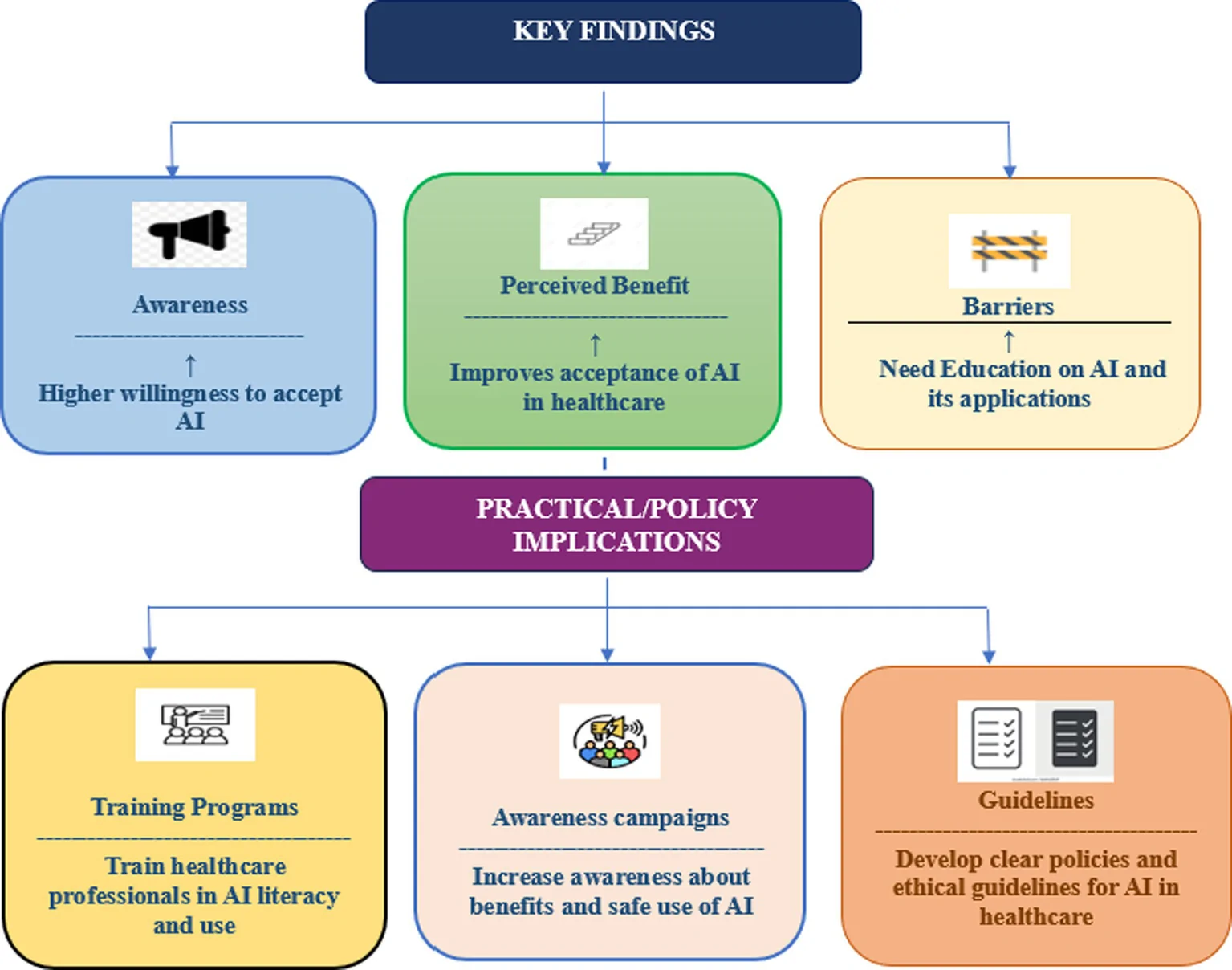
Summary of key findings and practical/policy implications regarding ChatGPT acceptance in healthcare.

The integration of artificial intelligence tools such as ChatGPT in healthcare communication presents important opportunities to enhance access to health information, support patient education, and complement healthcare services. However, this study identified concerns related to misinformation, data privacy, overreliance, and reduced human interaction, highlighting the importance of responsible implementation. Findings from this study indicate that awareness emerged as the strongest predictor of willingness to use ChatGPT, followed by trust and perceived usefulness, whereas risk perception showed a negative association. These results suggest that interventions focusing on improving awareness and strengthening trust and perceived usefulness are likely to be more effective in increasing acceptance than demographic-based strategies alone. Based on these findings, policy and practice initiatives should prioritize structured AI literacy programs to improve public understanding of generative AI tools. In addition, enhancing transparency, explainability, and reliability of AI systems is expected to strengthen trust and perceived usefulness among users. Furthermore, regulatory and institutional strategies addressing misinformation, data privacy, and data security, including formal AI governance frameworks, may help reduce perceived risks and support informed and safe engagement with AI technologies. Equity-oriented strategies, including accessible education campaigns and continuous monitoring of user perceptions, may support broader acceptance across diverse population groups. Aligning implementation strategies with these determinants may facilitate responsible adoption of ChatGPT in healthcare communication while maintaining public confidence, ethical standards, and system-level governance of AI deployment in healthcare settings. A summary of key findings and practical/policy implications regarding ChatGPT acceptance in healthcare is shown in Figure 1.

### 4.2 Limitations and future research

This study has several limitations that should be considered. First, the cross-sectional design limits the ability to assess changes in public perceptions over time and prevents causal inferences, meaning that observed associations reflect correlations rather than directional relationships. Second, data were collected through a self-administered online survey, which may introduce self-report bias and social desirability effects. In addition, the online format may have preferentially included participants with higher digital literacy, potentially underrepresenting individuals with limited internet access or lower familiarity with AI tools. Furthermore, participants were recruited using a non-probability convenience sampling approach through social media platforms, which may have introduced selection bias. Individuals with greater access to digital technologies, higher levels of digital literacy, and a stronger interest in technology-related topics may have been more likely to participate. Given that the study focused on perceptions of ChatGPT and AI in healthcare, respondents with prior exposure to AI tools or greater technological familiarity may have been overrepresented. The online recruitment strategy may also have contributed to non-response bias, as individuals who chose not to participate may differ systematically from those who completed the survey. In addition, the sample contained a higher proportion of male and urban participants, which may limit its representativeness of the broader population. Consequently, older adults, rural residents, and individuals with limited internet access may be underrepresented, thereby limiting the generalizability of the findings to the wider population of the Asir Region and Saudi Arabia. Third, although the sample included participants across diverse ages, educational levels, and occupations, including healthcare professionals, detailed subgroup analyses were not performed, such as comparisons between healthcare and non-healthcare participants or based on prior AI exposure. Additionally, the structured questionnaire format may have limited the capture of nuanced responses regarding trust, perceived usefulness, and ethical concerns.

Although probability-based sampling could have improved representativeness, it was not feasible in the present study because of the lack of a comprehensive sampling frame for the target population, the geographically dispersed nature of the community, and logistical constraints associated with large-scale population-based recruitment. Therefore, online convenience sampling was selected as a practical approach to facilitate timely community-level assessment of public perceptions regarding an emerging healthcare technology. Future research could adopt longitudinal designs to track changes in awareness, trust, and willingness to use AI healthcare tools over time. Qualitative studies may provide richer insights into ethical considerations, adoption barriers, and public perspectives. Expanding research to other regions or countries would enhance generalizability and enable cross-cultural comparisons of AI trust and adoption behaviors. Furthermore, future work could explore healthcare professionals’ perspectives, compare them with the general public, and incorporate objective behavioral data, such as real-time AI usage analytics, to complement self-reported measures. Future studies should also consider probability-based sampling strategies and, where appropriate, weighting procedures to improve population representativeness and reduce sampling bias. Finally, interventions such as educational programs or awareness campaigns could be evaluated for their associations with AI literacy, trust, and perceived risk levels, as well as their role in supporting informed decision-making regarding AI use in healthcare.

## 5 Conclusion

This study provides insight into public awareness, trust, perceived usefulness, and willingness to use ChatGPT in healthcare communication among adults in the Asir region of Saudi Arabia. Awareness of ChatGPT and AI chatbots was generally high, although formal education or training in AI was limited. Participants generally perceived ChatGPT as useful for improving access to health information and supporting patient education, while trust and perceived usefulness remained generally positive, although trust in its accuracy and safety was moderate and was accompanied by concerns regarding misinformation, privacy, and overreliance. Associational analyses indicated that higher awareness and trust were associated with higher willingness to use ChatGPT, whereas stronger perceptions of risk were associated with lower willingness to use ChatGPT. Differences observed across age and educational groups suggest variation in familiarity and confidence in AI technologies within the population. Overall, the findings reflect cautious public openness toward AI-enabled healthcare tools and highlight the need for education, transparent communication, and appropriate regulatory oversight to support informed engagement. Further research is warranted to monitor how public perceptions evolve as AI applications in healthcare continue to expand. Strengthening AI literacy, trust-building initiatives, and regulatory oversight may facilitate the responsible adoption of generative AI technologies in healthcare settings.

## Data Availability

The original contributions presented in the study are included in the article/Supplementary material, further inquiries can be directed to the corresponding author/s.
