# Supplementary material for: Public awareness, trust, perceived usefulness, and willingness to use ChatGPT for healthcare communication among adults in the Asir Region, Saudi Arabia: a cross-sectional study
Source: Front Public Health. 2026 Aug 21;14:1862006. doi: 10.3389/fpubh.2026.1862006 (PMC13541769; doi:10.3389/fpubh.2026.1862006)
Supplement: Supplementary file 1 [file Table_1.DOCX]

**Supplementary File**

**Effect sizes**

| **Variable** | **Outcome** | **Test** | **p-value** | **Effect Size** |
| --- | --- | --- | --- | --- |
| Age Group | Awareness | ANOVA | 0.001 | η² = 0.061 |
| Age Group | Trust | ANOVA | <0.001 | η² = 0.074 |
| Age Group | Willingness | ANOVA | 0.030 | η² = 0.034 |
| Education | Awareness | ANOVA | 0.030 | η² = 0.041 |
| Education | Trust | ANOVA | 0.030 | η² = 0.038 |
| Education | Willingness | ANOVA | 0.092 | η² = 0.018 |
| Gender | Awareness | Independent t-test | 0.412 | Cohen's d = 0.08 |
| Gender | Trust | Independent t-test | 0.365 | Cohen's d = 0.06 |
| Gender | Willingness | Independent t-test | 0.488 | Cohen's d = 0.07 |
| Residence | Awareness | Independent t-test | 0.281 | Cohen's d = 0.11 |
| Residence | Trust | Independent t-test | 0.327 | Cohen's d = 0.09 |
| Residence | Willingness | Independent t-test | 0.194 | Cohen's d = 0.14 |
| Prior ChatGPT Use | Awareness | Independent t-test | <0.001 | Cohen's d = 0.67 |
| Prior ChatGPT Use | Trust | Independent t-test | <0.001 | Cohen's d = 0.59 |
| Prior ChatGPT Use | Willingness | Independent t-test | <0.001 | Cohen's d = 0.73 |

**Regression Assumption Testing and Model Quality Indicators**

| **Assumption/Diagnostic** | **Method** | **Result** |
| --- | --- | --- |
| Normality of residuals | Shapiro-Wilk | p = 0.087 |
| Independence of errors | Durbin-Watson | 1.94 |
| Multicollinearity | VIF range | 1.12–1.64 |
| Tolerance | Range | 0.61–0.89 |
| Overall model fit | F (6,422) | 12.87 |
| Explained variance | Adjusted R² | 0.167 |
